# Optical Coherence Tomography-Guided Transepithelial Phototherapeutic Keratectomy for Central Corneal Opacity in the Pediatric Population

**DOI:** 10.1155/2018/3923617

**Published:** 2018-12-24

**Authors:** Sloan W. Rush, Ryan B. Rush

**Affiliations:** ^1^Panhandle Eye Group, 7400 Fleming Ave., Amarillo, TX 79106, USA; ^2^Texas Tech University Health Science Center, 1400 S. Coulter, Amarillo, TX 79106, USA; ^3^Southwest Retina Specialists, 7411 Wallace Blvd., Amarillo, TX 79106, USA

## Abstract

**Purpose:**

To report the outcomes of optical coherence tomography- (OCT-) guided transepithelial phototherapeutic keratectomy (PTK) for central corneal opacity in the pediatric population.

**Methods:**

The charts of 10 eyes of 8 children aged 9 to 17 with central corneal opacity from various pathologies who underwent a standardized OCT-guided transepithelial PTK technique at a single private practice institution were retrospectively reviewed. The corneal topographic findings, OCT measurements, and visual results with refractive outcomes were analyzed 6 months after the PTK treatment.

**Results:**

All 10 eyes tolerated the procedure well without any significant intraoperative or postoperative complications. Uncorrected and best spectacle-corrected visual acuity (BSCVA) significantly improved postoperatively (*p* < 0.0001 and *p*=0.0045, respectively). The absolute value of spherical equivalent on cycloplegic refraction significantly improved postoperatively as well (*p*=0.0014), but there were no significant changes in topographic measurements. Seven out of the 10 eyes had complete resolution of the central corneal opacity on OCT imaging. None of the subjects lost any lines of BSCVA and developed recurrence of the corneal opacity from the primary disease condition or required keratoplasty during the follow-up period.

**Conclusions:**

OCT-guided transepithelial PTK can provide excellent visual outcomes in pediatric patients with central corneal opacities.

## 1. Introduction

Corneal opacities can arise from a number of pathological conditions including congenital diseases [1] and dystrophies [2], trauma [3], limbal stem cell deficiencies [4], corneal degenerations [5], trachoma [6], herpetic corneal disease [7], and other corneal infections [8]. Surgical strategies to treat corneal opacities range from invasive options with penetrating [9] and lamellar keratoplasty [10] to minor, in-office procedures with superficial keratectomy [11] or excimer laser photoablation [12]. While corneal transplantation remains a viable option for the adult population [13], it still poses significant risk in children [14].

Phototherapeutic keratectomy (PTK) for the treatment of corneal pathology dates back to the introduction of the excimer laser [15, 16] with over 25 years of clinical use [17]. In adults, PTK has been used extensively for the treatment of corneal scarring, corneal dystrophies/degenerations, and recurrent corneal erosions [18, 19]. These various PTK techniques have used broad-beam ablations [19], intraoperative masking agents [20], focal [21] and selective zonal [22] ablations, dual ablations [23], transepithelial methods [24], wavefront-guided ablations [25], topography-guided ablations [26], and wavefront-optimized ablations [27]. However, there are relatively few studies in which any PTK technique has been reported for its use in the pediatric population [28–32], especially using more advanced techniques which take the advantage of novel imaging modalities.

Recent studies among adults have shown that optical coherence tomography (OCT) has improved preoperative assessment and demonstrated effectiveness in guiding PTK techniques when treating corneal opacities associated with irregular astigmatism [33, 34], and predictable postoperative refractive outcomes may be achieved when an OCT-measured depth of treatment calculation is combined with a transepithelial approach [24, 35]. Presently, there are no reports describing the OCT-guided transepithelial PTK technique for the treatment of central corneal opacification in the pediatric population.

## 2. Methods

The SRS Institutional Review Board (IRB00009122) approved this retrospective case series of pediatric patients who underwent OCT-guided transepithelial PTK for the treatment of visually significant central corneal opacification from November 2011 through November 2017 at a single private practice institution in Amarillo, TX. All components of the study adhered to the tenets of the Declaration of Helsinki and were performed in accordance with human research standards and regulations.

Consecutive pediatric patients who underwent OCT-guided transepithelial PTK using the Wavelight EX500 excimer laser platform (Alcon, Fort Worth, TX, USA) for the treatment of visually significant central corneal scarring were included. Patients were considered pediatric if they were <18 years at the time of PTK. The corneal scarring was considered visually significant when the best spectacle-corrected visual acuity (BSCVA) was worse than Snellen 20/40 and was, in the opinion of the examiner, responsible for at least 2 Snellen lines of reduced visual acuity. The opacity was considered central when it was located within 3 mm of the pupillary center of the cornea on slit lamp examination. An irregular Bowman's layer was the defining feature in all cases with corneal scarring. An irregular Bowman's layer was considered to be present when spectral domain OCT (Cirrus HD-OCT; Carl Zeiss Meditec, Inc, Dublin, California, USA) was observed to have a hyperintense signal that corresponded to the central corneal opacity seen on clinical examination and was associated with an epithelial thickness variation by a minimum of a 33% increase from the baseline epithelial thickness.

The demographic and preoperative data collected at baseline from each subject included age, gender, operative eye, underlying etiology of corneal opacity, other existing ocular comorbidities, uncorrected visual acuity (UCVA), BSCVA, spherical equivalent (SE) on cycloplegic refraction, refractive astigmatism on cycloplegic refraction, and corneal topographic-measured cylinder, surface asymmetry index (SAI), surface regularity index (SRI), and projected visual acuity (PVA) using the TMS-4N Topographer (Tomey; Phoenix, AZ, USA). Charts were reviewed for any intraoperative or postoperative complication occurring during the study period. The UCVA, BSCVA, SE on cycloplegic refraction, refractive astigmatism on cycloplegic refraction, and corneal topography measurements were collected at 6-month (±2 months) follow-up after the PTK treatment. JMP 11 software from the SAS Institute (Cary, NC, USA) was used to analyze distributions and calculate means with standard deviations. One-way analysis of the variance was used to compare the means of the baseline measurements with the post-PTK measurements. Visual acuity change was considered significant if there was an improvement by logMAR 0.3 or more, whereas the other comparisons were considered statistically significant at the alpha <0.05 level.

### 2.1. Phototherapeutic Keratectomy Technique

**Figure 1:**
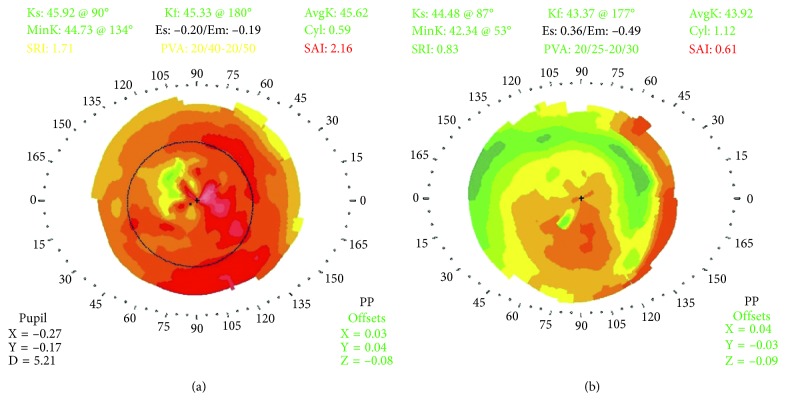
Optical coherence tomography-guided transepithelial phototherapeutic keratectomy for central corneal opacity in the pediatric population. Corneal topography before and after phototherapeutic keratectomy. (a) Preoperative corneal topography of a 9-year-old female who developed corneal scarring after an episode of herpes simplex virus keratitis. Central irregularity is quite apparent. The preoperative best spectacle-corrected visual acuity was 20/80. (b) Corneal topography of the same patient from A 6 months after phototherapeutic keratectomy. There has been an improvement in central corneal regularity and in the topographic indices. The best spectacle-corrected visual acuity improved to 20/30.

**Figure 2:**
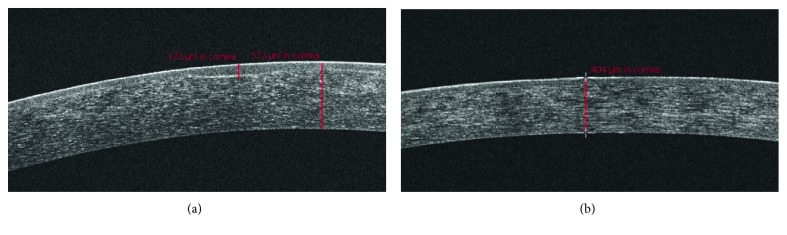
Optical coherence tomography-guided transepithelial phototherapeutic keratectomy for central corneal opacity in the pediatric population. Corneal optical coherence tomography before and after phototherapeutic keratectomy. (a) Preoperative optical coherence tomography of the same 9-year-old female in Figure 1. Notice the central corneal opacity with adjacent irregularity of Bowman's layer and epithelial compensation. A manual electronic caliper (red lines) measured the maximum depth of the corneal scar at 120 microns and total corneal pachymetry of 516 microns. Using this information, transepithelial phototherapeutic keratectomy calculations were made to treat to a target depth of 139 microns (which is expected to photoablate 91 microns of stroma after accounting for the measured baseline epithelial thickness of 48 microns) to preserve refractive neutrality. (b) Optical coherence tomography of the same patient from A 6 months after phototherapeutic keratectomy. After a predicted total stromal ablation depth of 91 microns, the central corneal opacity and irregularity in Bowman's layer is totally resolved with complete restoration in the uniformity of the epithelium. Central corneal pachymetry is now 404 microns after reepithelialization and wound contraction.

All pediatric subjects were cooperative enough to undergo PTK under topical anesthesia with proparacaine without sedation of any kind. All known or suspected cases of previous herpes simplex virus keratitis were pretreated with oral acyclovir (weight-dependent dosing) for one week prior to PTK and for 6 months after PTK. The same standardized OCT-guided transepithelial PTK technique described thoroughly in previous studies was used on all subjects [35, 36]. Briefly, OCT images of the cornea were used to measure the total thickness of the central cornea, baseline epithelial thickness, and the maximum depth of the corneal opacity. Using this data, standardized calculations were made to determine excimer laser treatment parameters including the depth of treatment in order to eliminate or reduce central corneal opacities while leaving at least 300 microns of residual stromal bed and simultaneously providing the desired spherical equivalent refractive outcome using a combined/consecutive myopic and hyperopic ablation. PTK was performed with a transepithelial approach including topical use of mitomycin C (0.02%) and application of a pair of bandage contact lenses at the conclusion of the treatment. Patients were treated postoperatively with topical ofloxacin 0.3% QID and topical prednisolone acetate 1% QID for 3 weeks. The pair of bandage contact lenses was removed after 5–7 days. Figures 1 and 2 provide an example of the technique used on one of the study subjects.

## 3. Results

There were 10 eyes of 8 children included in the analysis. The mean follow-up was 8.33 (±4.53) months after PTK. The baseline characteristics and demographic features of the study population are summarized in Table 1. The baseline UCVA was 1.70 (1.37–2.02) logMAR, while baseline BSCVA was 0.60 (0.43–0.77) logMAR. The preoperative spherical equivalent of the study population was −2.18 (±3.55) diopters with a range of −10.63 to +2.00 diopters. The preoperative average depth of the central corneal opacity was 135.2 (±74.4) microns with the minimum depth of 64 microns and maximum depth of 320 microns.

The target excimer laser ablation depth was 139.4 (±30.9) microns. There were no notable intraoperative complications associated with the PTK treatment. All patients were cooperative enough to safely perform the treatment, and the excimer laser was able to successfully track the pupil in both cases in which there was low frequency, low amplitude nystagmus.

The residual opacity depth on OCT of the central cornea after PTK was 22.2 (±46.6) microns. Seven out of the 10 treated eyes had complete resolution of the corneal opacity. UCVA and BSCVA significantly improved from the baseline level after treatment with PTK (*p* < 0.0001 and *p*=0.0045, respectively). Although there was a trend toward improvement in the topographic cylinder and SRI (*p*=0.09 for both), there were no significant changes in any of the topographic parameters analyzed. No patients lost any Snellen lines of BSCVA, and all patients gained at least one line of Snellen BSCVA after PTK. There were no cases of corneal opacity recurrence during the study interval. A summary of the outcomes after PTK are presented in Table 2.

With regards to the refractive outcomes, the target SE refractive goal for all 10 eyes was plano. Absolute value of the SE on cycloplegic refraction was significantly improved postoperatively (*p*=0.0014). The SE on cycloplegic refraction 6 months after PTK was 0.64 (±0.58) diopters, where 8 of the eyes had some degree of hyperopia and 2 of the eyes had some degree of myopia. No patients experienced postoperative anisometropia as defined by SE difference in 3 diopters or more between the eyes. Total refractive astigmatism on cycloplegic refraction 6 months after PTK was 1.67 (±1.26) diopters. No study on the eyes underwent further surgery with keratoplasty or repeated excimer laser refractive treatment during the study interval.

## 4. Discussion

To our knowledge, this is the first case series to report OCT-guided transepithelial PTK in the pediatric population. Previous PTK studies in children have used older excimer laser technology with broad-beam lasers from the 1990s [12, 15–17]. These techniques did not calculate optimal ablation depths preoperatively and often resulted in unpredictable amounts of hyperopia postoperatively [37]. The introduction of OCT imaging into the process gives the advantage of preoperatively determining opacity depth into the corneal stroma and measuring epithelial thickness variations adjacent to the opacity. These OCT measurements help in guiding the input for the excimer laser treatment parameters so that an appropriate depth of the treatment results [34]. In addition, a transepithelial PTK approach does not require the use of a masking agent in areas of the corneal opacity in which there are significant epithelial variability [24]. The OCT-guided transepithelial PTK technique used in this study resulted in excellent visual outcomes with substantial reduction or complete elimination of the central corneal opacity in the pediatric population studied; the technique was well-tolerated by the pediatric population and without significant intraoperative or postoperative complications.

Attaining desired postoperative refractive outcomes in the pediatric population is critical because large amounts of induced hyperopia may result in anisometropia, spectacle intolerance, and loss of binocular vision [38]. Children often can not tolerate contact lens therapy. The refractive outcomes in our study population were very predictable, and there were no eyes that had large refractive surprises or visually significant anisometropia after PTK. Although the visual outcomes improved tremendously after PTK, topographic irregularities persisted to some degree and did not significantly improve after the surgery. However, the authors did notice a statistical trend toward improvement in the SRI which may have shown significance with a higher number of patients. These findings suggest that the elimination of the physical opacity on the cornea may have a greater impact on visual acuity than corneal topography changes after PTK in the pediatric population, which is likely to have a different healing response to treatment when compared to the adult population.

Weaknesses of this study include its retrospective study design, the lack of a control group, the small number of cases, and the relatively short follow-up interval. In particular, the short follow-up period of this study is inadequate to determine if the pediatric population is at higher risk for corneal haze than the adult population. Children younger than 8 or 9 years old are not likely able to cooperate well enough to perform excimer laser photoablation under topical anesthesia, so caution should be used when applying the results of this study to the youngest of the pediatric population. General anesthesia would be necessary to assess the PTK technique described in this study in those patients. Future prospective investigations will be needed to validate and compare the PTK technique described in this study with previously described broad-beam laser PTK techniques in the pediatric population.

## Figures and Tables

**Table 1:** Optical coherence tomography-guided transepithelial phototherapeutic keratectomy for central corneal opacity in the pediatric population. Baseline characteristics and demographic features of the study population.

Preoperative characteristics and demographics (*n*=10 eyes)	Means (standard deviations)
Age (years)	14.0 (3.0), range = 9 to 17
Gender	90% female and 10% male
Operative eye	60% right eye and 40% left eye
Underlying pathology for central corneal opacity	HSV keratitis: *n*=4 (40%)
	Contact lens-related bacterial keratitis: *n*=2 (20%)
	Contact lens-related fungal keratitis: *n*=2 (20%)
	Anterior corneal dystrophy: *n*=2 (20%)
Comorbidities	Congenital motor nystagmus: *n*=2 (20%)
	Mild amblyopia: *n*=2 (20%)
	Soft contact lens war: *n*=2 (40%)

**Table 2:** Optical coherence tomography-guided transepithelial phototherapeutic keratectomy for central corneal opacity in the pediatric population. Visual and anatomic outcomes after phototherapeutic keratectomy.

Outcomes (*n* = 10 eyes)	Preoperative means (95% confidence intervals)	Postoperative means (95% confidence intervals)	*p* value
Uncorrected visual acuity (logMAR)	1.70 (1.37–2.02), range = 0.65–2.5	0.45 (0.12–0.78), range = 0.00–0.70	<0.0001
Best spectacle-corrected visual acuity (logMAR)	0.60 (0.43–0.77), range = 0.2–1.2	0.21 (0.03–0.39), range = 0.00–0.54	0.0045
Topographic cylinder	4.47 (2.62–6.31), range = 0.76–13.83	2.24 (0.29–4.19), range = 0.72–3.33	0.0989
Topographic surface asymmetry index	1.76 (0.85–2.66), range = 0.19–6.47	1.01 (0.11–1.91), range = 0.37–3.01	0.2362
Topographic surface regularity index	1.18 (0.80–1.56), range = 0.25–1.98	0.72 (0.34–1.10), range = 0.03–1.59	0.0924
Topographic projected visual acuity (logMAR)	0.44 (0.36–0.52), range = 0.00–0.44	0.42 (0.34–0.50), range = −0.05–0.35	0.6991
Refractive astigmatism on cycloplegic refraction (diopters)	1.73 (0.96–2.49), range = 0.25–3.75	1.67 (0.86–2.47), range = 0.75–4.50	0.9130
Absolute value of spherical equivalent on cycloplegic refraction (diopters)	4.71 (3.17–6.26), range = 1.00–10.63	0.67 (−0.97–2.30), range = 0.13–2.00	0.0014

## Data Availability

The data for this study are available upon request.

## Abbreviations

OCT: Optical coherence tomography
PTK: Phototherapeutic keratectomy
BSCVA: Best spectacle-corrected visual acuity
UCVA: Uncorrected visual acuity.
